# Case Report: Giant left atrial cystic tumor: myxoma or intracardiac blood cyst?

**DOI:** 10.3389/fcvm.2024.1323890

**Published:** 2024-02-14

**Authors:** Weizhang Xiao, Jing Qin, Jia Feng, Feng Jiang, Xinming Chen, Xiang Cao, Qun Xue, Jiahai Shi

**Affiliations:** ^1^ Department of Cardiothoracic Surgery, Affiliated Hospital and Medical School of Nantong University, Nantong, China; ^2^ Department of Echocardiography, Affiliated Hospital and Medical School of Nantong University, Nantong, China; ^3^ Department of Pathology, Affiliated Hospital and Medical School of Nantong University, Nantong, China

**Keywords:** case report, cardiac tumor, myxoma, intracardiac blood cyst, cardiac surgery

## Abstract

**Background:**

Primary cardiac tumors are uncommon, with the majority being benign myxomas. Cystic myxoma, a particularly rare type of benign cardiac tumor, demands cautious differential diagnosis from other cardiac tumors.

**Case summary:**

A 43-year-old male patient presenting with intermittent dyspnea was referred to our department for surgical evaluation. Transthoracic echocardiography (TTE) and transesophageal echocardiography (TEE) unveiled an intra-left atrial cyst, which was subsequently found to be blood-filled during a video-assisted microinvasive heart surgery. Pathological examination depicted a cyst wall filled with small stellate and fat spindle cells, along with a mucoid matrix, indicating a diagnosis of cystic myxoma.

**Conclusions:**

We herein presented a rare case of an adult patient with cystic myxoma, initially misdiagnosed as an intracardiac blood cyst (CBC) prior to surgery, and ultimately verified via pathological findings.

## Introduction

Primary cardiac tumors are rare, with an incidence of 0.002% to 0.3% and a prevalence of 0.001% to 0.03%. Over half of these tumors are myxomas (1). Most myxomas occur in the left atrium (75% to 80%), with fewer cases found in the right atrium (10% to 20%) (2). Typically, these tumors are solid, round, or polypoid in shape and attached to the interatrial septum, lacking a cystic structure. However, cystic myxomas are exceedingly rare (3–8). Intracardiac blood cysts are another unusual cardiac tumor, mainly seen in fetuses or infants under six months of age and rarely reported in adults. Here, we present an uncommon case of a left atrial cystic myxoma that was initially suspected as a CBC before surgery and ultimately diagnosed as a cystic myxoma.

## Patient information

**Figure 1:**
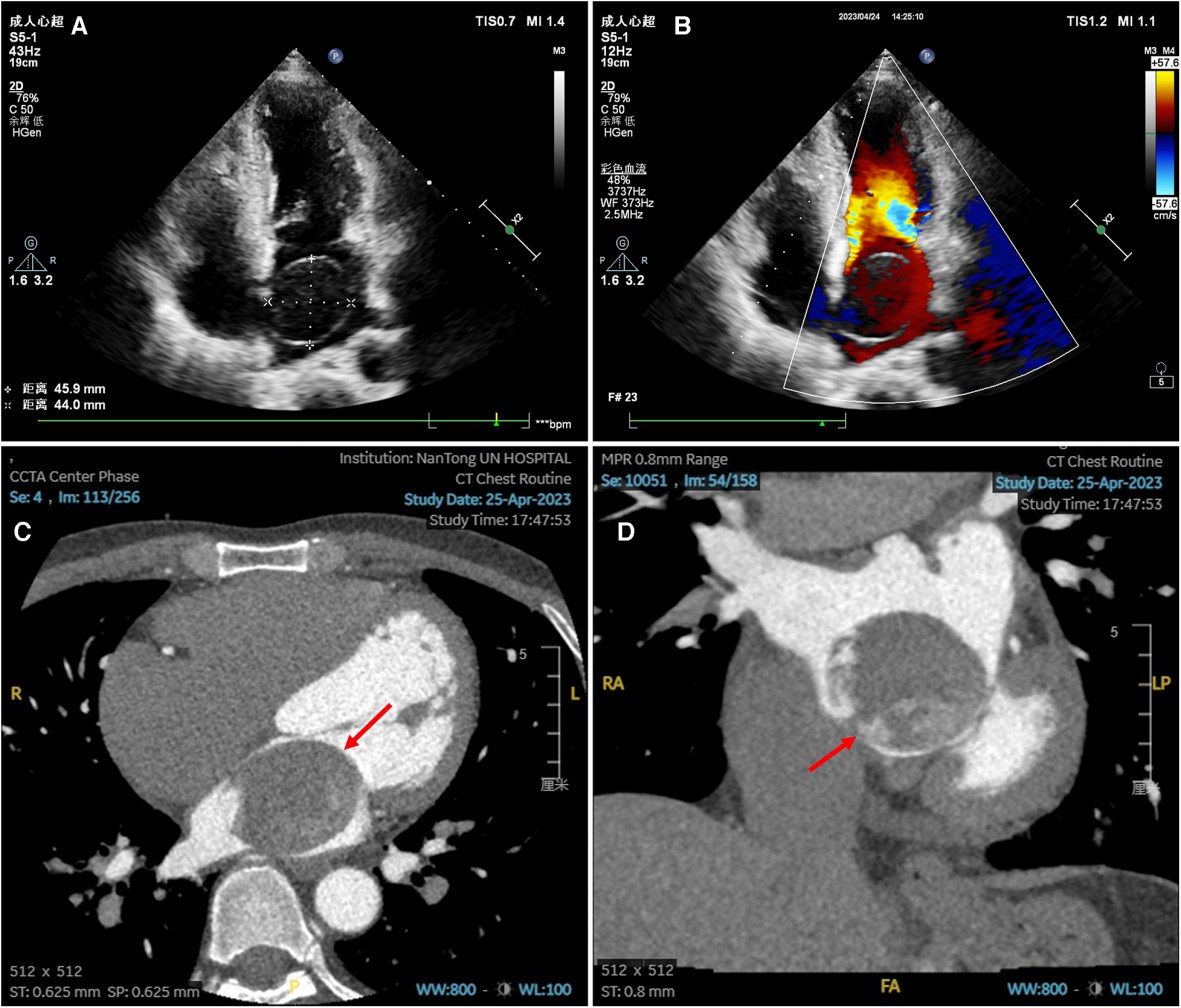
Preoperative echocardiography and enhanced cardiac CT scan images. (**A–B**) Transthoracic echocardiography showed an echoless tumor measuring 4.6 × 4.4 cm in the left atrium, with clear boundary, a small base attached to the atrial septum, and no blood signal in the tumor. (**C–D**) Enhanced cardiac CT scan in transverse plane (**C**) and coronal plane (**D**) showed a giant tumor in the left atrium with heterogeneous density after enhancement.

A 43-year-old male patient presented with intermittent dyspnea during sleep for 20 days and underwent TTE at a local hospital, which revealed a left atrial myxoma. He was referred to our hospital for surgical evaluation and was further directed to the cardiothoracic surgery department. The patient denied any history of hypertension, diabetes mellitus, and tobacco use. Upon physical examination, a moderate diastolic murmur was auscultated at the cardiac apex area. Electrocardiography showed a sinus rhythm with a heart rate of 63 beats per minute. All initial laboratory tests were within normal limits, except for a mild elevation in B-type natriuretic peptide (108 pg/L). Additionally, hemoglobin was 143 g/L, albumin was 38.5 g/L, aspartate aminotransferase was 13 U/L, alanine aminotransferase was 12 U/L, erythrocyte sedimentation rate was 2 mm/hour, and Troponin was 0.011 μg/L. TTE demonstrated an almost echoless mass measuring 4.6 × 4.4 cm with well-defined margins in the left atrium, which was attached to the atrial septum with a narrow base (Figures 1A,B). TEE showed a large cystic mass in the left atrium that protruding into the mitral valve during diastole, causing mild mitral stenosis (Supplementary Video S1). A cardiac CT scan displayed a giant tumor located in the left atrium with heterogeneous density following enhancement, suggesting the possibility of a myxoma (Figures 1C,D).

**Figure 2:**
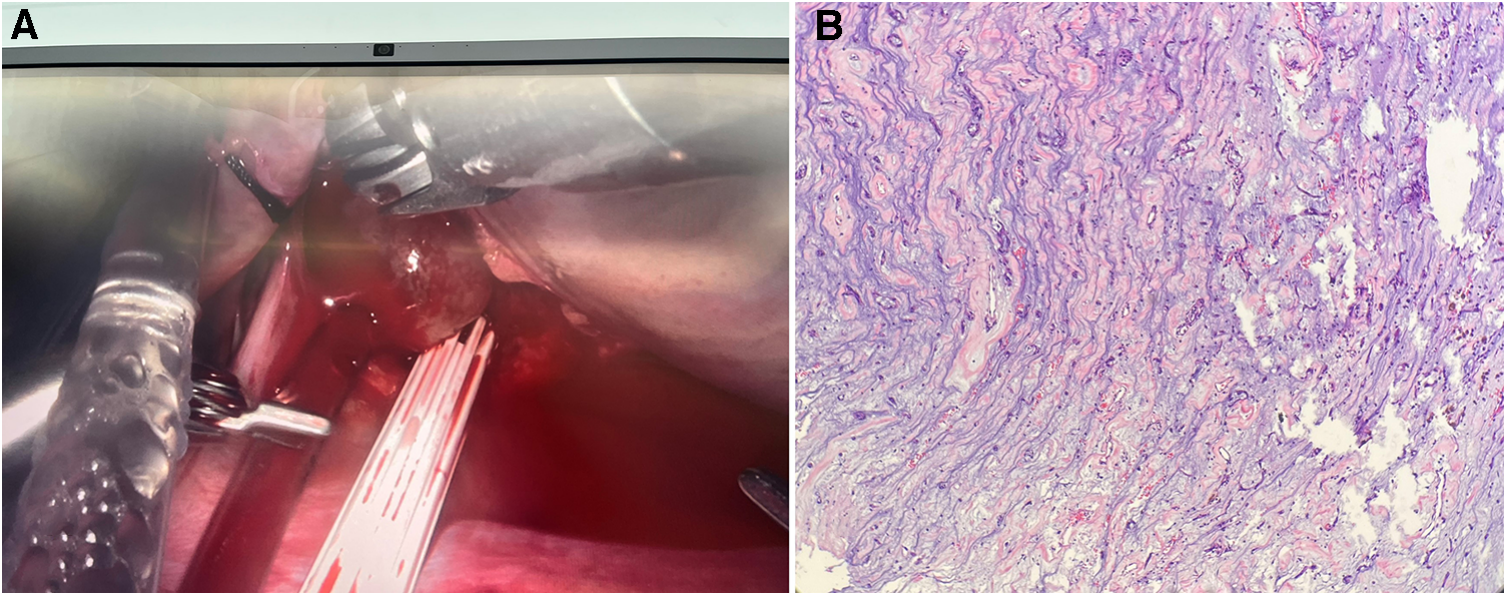
Intraoperative and pathological images. (**A**) Intraoperative view revealed a parenchymal blood-filled cyst with smooth surface attached to the atrial septum with a pedicle. (**B**) Pathological examination of the cyst (hematoxylin and eosin stain, ×4) showed spindle cells with eosinophilic cytoplasm surrounded by mucoid matrix.

The patient underwent a video-assisted thoracic surgery using the Da Vinci surgical system (Intuitive Surgical Inc., Sunnyvale, CA, USA). After double-lumen intubation, cardiopulmonary bypass (CPB) was established by femoral artery and vein cannulation, and a right-sided incision was made to access the chest cavity. During the surgery, a giant parenchymal cyst was found in the left atrium with a pedicle attached to the atrial septum, which was intact (Figure 2A). We attempted to completely remove this lesion. Unfortunately, the cyst accidentally ruptured during resection, causing blood to flow out and resulting in its collapse. Nevertheless, the tumor was completely removed without causing any damage to the adjacent tissue. The total operation time was 2 h and 14 min, the CPB time was 1 h and 18 min, and the cross-clamp time was 35 min. Pathological examination revealed a smooth cyst wall with a thickness of 0.1–0.2 cm. The cyst wall consisted of small stellate and fat spindle cells with round, oval, or elongated nuclei and eosinophilic cytoplasm, surrounded by a mucoid matrix. The tumor cells were arranged in a linear pattern and oriented towards the blood vessels, with no cytological atypia present (Figure 2B). The postoperative recovery process was uneventful, and the patient was transferred from the intensive care unit to the general ward on the second day after surgery, and discharged nine days later, with normal mitral valve and left ventricular functions confirmed through echocardiography before discharge. During the follow-up period, there was no recurrence of myxoma.

## Discussion

As a very rare tumor, the pathological type of cardiac mass is closely related to its location. For instance, myxoma can always be seen in the left atrium, lipoma is more common in the right atrium or left ventricle, and fibroma and rhabdomyoma are more common in the ventricles (9). While most diagnoses of cardiac tumors are rendered through imaging techniques, some of them may not exhibit characteristic imaging features that suggest their pathological type, making the diagnosis challenging. Therefore, myocardial biopsy plays an important role in the diagnosis of cardiac tumors. Currently, computerized tomography (CT), TEE, or intracardiac echocardiography are commonly used to guide myocardial biopsy (10). Among them, CT-guided biopsy has been proven to be safe for pericardial or superficial intermural tumors (11). For intracardiac masses, myocardial biopsy is most performed via the venous route, including internal jugular or femoral vein, and for left ventricular lesions, biopsy can also be performed by transseptal puncture or directly through peripheral arteries (12). Considering the high risk of myocardial biopsy including vasovagal reaction, pericardial tamponade, arrhythmias, ventricular perforation, and vascular injury (13), it is generally utilized for tumors in the right cardiac system (14). Additionally, myocardial biopsy is considered contraindicated in the following situations: (1) cardiac tumors without a safe puncture path; (2) the surface of mass is surrounded by large tortuous blood vessels; (3) preoperative imaging showing significant necrosis within the lesion or severe coagulation dysfunction; (4) severe dyspnea or agitation; and (5) friable masses with a high embolic potential, such as left ventricular tumors or typical cardiac myxomas (11).

As the most common cardiac tumor, the clinical features of cardiac myxoma depend on the location and size of tumor. Dyspnea is the most frequent symptom of left atrial myxoma, which is a characteristic manifestation of mitral valve dysfunction leading to left-sided heart failure. Conversely, myxoma in the right atrium may result in symptoms related to right-sided heart failure. Given that the majority of cardiac myxomas exhibit a non-cystic structure, embolism should be of particular concern, with approximately half of all cases presenting embolism-related events, including strokes, retinal artery emboli, and limb embolism (15).

Cystic myxoma is a relatively rare condition that is typically identified incidentally via TTE. Through searching PubMed, only a few literatures were found to report the existence of cystic myxoma as shown in Table 1. Reports have indicated the presence of feeding arteries into the tumor, originating from either the left or right coronary artery, potentially leading to coronary steal phenomenon such as angina (5, 8). We hypothesize that hemorrhage within the tumor contributes to the formation of a cystic mass filled with blood. Moreover, the stability of this blood-filled cyst requires a sufficient drainage hole for outlet flow. Diagnosing cystic myxoma requires caution, as the differential diagnosis includes several other conditions, such as CBC, hemangioma, angiosarcoma, hydatid cyst, thrombus, and metastatic tumors (8, 21).

**Table 1:** Reported cases of cystic cardiac myxoma in the literature till January 2024.

Numbers	Author, year	Age	Gender	Chief complaint	Location	Size
1	Okuri H. (16)	54	Male	Dyspnea	Right atrium	69 × 44 mm
2	Lee KT. (17)	72	Female	Dyspnea	Left atrium	30 × 30 mm
3	Benezet-Mazuecos J. (18)	59	Female	Dyspnea	Left atrium	55 × 30 mm
4	Park J. (19)	71	Male	Dyspnea	Left atrium	53 × 32 mm
5	Acikel S. (5)	38	Female	Dyspnea, angina	Left atrium	75 × 50 mm
6	Park, KJ. (6)	65	Female	Fever, malaise	Left atrium	24 × 23 mm
7	Toprak, C. (7)	47	Male	Dyspnea	Left atrium	58 × 38 mm
8	Liao JM. (20)	35	Female	Dyspnea	Left atrium	70 × 50 mm
9	Watanabe H. (21)	75	Male	Dyspnea	Left atrium	54 × 39 mm
10	Shabestari MM. (22)	69	Female	Dyspnea, chest pain	Left atrium	26 × 25 mm
11	Xie, X. (4)	62	Female	Dyspnea	Left atrium	35 × 30 mm
12	Ntinopoulos V. (3)	63	Female	Dyspnea	Left atrium	29 × 22 mm
13	Suzuki T. (23)	44	Male	Dyspnea	Left atrium	N/A
14	Azad S. (24)	11	Male	Chest and throat pain	Left ventricle	39 × 26 mm
15	Futami S. (8)	73	Male	N/A	Left atrium	32 × 24 mm

CBC, first documented in 1,844, remains a relatively uncommon benign cardiac tumor, primarily discovered during autopsy and rarely reported in adults (25). Many patients with CBC exhibit no symptoms and are often diagnosed incidentally during routine echocardiography. Nevertheless, CBCs positioned in the semilunar or atrioventricular valves, as is most commonly the case, may induce valve dysfunction.

In the present case, given the smooth and thin morphology of the cyst wall, a CBC was strongly suspected prior to surgery. This suspicion was further reinforced by the intraoperative observations, which revealed a blood-filled, balloon-like cyst. Although an enhanced cardiac CT scan failed to demonstrate any feeding arteries within the tumor, histopathological examination was crucial for arriving at a definitive diagnosis. The result revealed a cyst wall exhibiting myxoma manifestations and lacking endothelial structure, confirmatively indicating a cystic myxoma.

## Conclusion

In this case report, we present an extremely rare case of a cystic left atrial myxoma. Preoperative echocardiography of the patient revealed a thin-walled cyst, which was further confirmed during surgery. Additionally, the cystic fluid was found to be bloody, leading us to initially suspect it as a CBC. However, upon the histopathological examination of the tissue, it was conclusively diagnosed as a cystic myxoma rather than CBC due to the absence of endothelial structure in the cyst wall and the presence of distinctive histological features of myxoma.

## Data Availability

The original contributions presented in the study are included in the article/Supplementary Material, further inquiries can be directed to the corresponding author.

## References

[1] Rahouma M Arisha MJ Elmously A El-Sayed Ahmed MM Spadaccio C Mehta K Cardiac tumors prevalence and mortality: a systematic review and meta-analysis. Int J Surg. (2020) 76:178–89. 10.1016/j.ijsu.2020.02.039 32169566

[2] Layton S Ripley DP Bellenger NG. Left atrial myxoma. Br Med J. (2013) 347:f4430. 10.1136/bmj.f4430 23894179

[3] Ntinopoulos V Dushaj S Brugnetti D Rings L Loeblein H Dzemali O. Left atrial myxoma: unusual presentation as a cystic tumor. J Card Surg. (2020) 35:511–3. 10.1111/jocs.14401 31856315

[4] Xie X Bai J. Left atrial myxoma presenting as a cystic mass. J Card Surg. (2017) 32:694–5. 10.1111/jocs.13224 28967159

[5] Acikel S Aksoy MM Kilic H Karapinar K Oguz AS Aydin H Cystic and hemorrhagic giant left atrial myxoma in a patient presenting with exertional angina and dyspnea. Cardiovasc Pathol. (2012) 21:e15–8. 10.1016/j.carpath.2011.01.006 21397522

[6] Park KJ Woo JS Park JY. Left atrial myxoma presenting with unusual cystic form. Korean J Thorac Cardiovasc Surg. (2013) 46:362–4. 10.5090/kjtcs.2013.46.5.362 24175272 PMC3810559

[7] Toprak C Kahveci G Tabakci MM Acar G Emiroglu MY. Unusual image of a cystic atrial myxoma: mass in mass appearance in the left atrium. Herz. (2015) 40:259–60. 10.1007/s00059-013-3923-y 23912972

[8] Futami S Hieda M Fukata M Shiose A. A rare case of cardiac myxoma with light bulb-like cystic morphology: a case report. Eur Heart J Case Rep. (2023) 7:ytad331. 10.1093/ehjcr/ytad331 37547377 PMC10398420

[9] Seferović PM Tsutsui H McNamara DM Ristić AD Basso C Bozkurt B Heart failure association of the ESC, heart failure society of America and Japanese heart failure society position statement on endomyocardial biopsy. Eur J Heart Fail. (2021) 23:854–71. 10.1002/ejhf.2190 34010472

[10] Naruse G Kawasaki M Yanase K Tanaka T. Primary angiosarcoma in the right atrium diagnosed by a cardiac tumor biopsy using intracardiac echocardiography. J Med Ultrasound. (2020) 28:120–2. 10.4103/JMU.JMU_93_19 32874873 PMC7446697

[11] Xie Y Hong ZL Zhao YC Chen S Lin YC Wu SS. Percutaneous ultrasound-guided core needle biopsy for the diagnosis of cardiac tumors: optimizing the treatment strategy for patients with intermural and pericardial cardiac tumors. Front Oncol. (2022) 12:931081. 10.3389/fonc.2022.931081 35992842 PMC9389083

[12] Veinot JP. Diagnostic endomyocardial biopsy pathology–general biopsy considerations, and its use for myocarditis and cardiomyopathy: a review. Can J Cardiol. (2002) 18:55–65. PMID: 11826329 11826329

[13] Oliveira GH Al-Kindi SG Hoimes C Park SJ. Characteristics and survival of malignant cardiac tumors: a 40-year analysis of >500 patients. Circulation. (2015) 132:2395–402. 10.1161/CIRCULATIONAHA.115.016418 26467256

[14] Veinot JP. Endomyocardial biopsy–when and how. Cardiovasc Pathol. (2011) 20:291–6. 10.1016/j.carpath.2010.08.005 20934890

[15] Butany J Nair V Naseemuddin A Nair GM Catton C Yau T. Cardiac tumours: diagnosis and management. Lancet Oncol. (2005) 6:219–28. 10.1016/S1470-2045(05)70093-0 15811617

[16] Okuri H Shimizu M Yokoyama K Kawada H Irisawa A Kikawada R. A case of right atrial myxoma: M-mode and pulsed-doppler echocardiographic findings before and after operation. Kokyu to Junkan. (1993) 41:397–401. PMID: 8516580 8516580

[17] Lee KT Lai WT Yen HW Voon WC Hwang CH Lu YH Cystic left atrium myxoma–a rare case report. Kaohsiung J Med Sci. (2001) 17:579–81. PMID: 11852466 11852466

[18] Benezet-Mazuecos J Marcos-Alberca P Farre J Manzarbeitia F Rabago R Rey M. Multicystic/cavitated giant left atrial myxomas: a matter of technology. Eur J Echocardiogr. (2008) 9:101–2. 10.1016/j.euje.2007.03.040 17588502

[19] Park J Song JM Shin E Jung SH Kim DH Kang DH Cystic cardiac mass in the left atrium: hemorrhage in myxoma. Circulation. (2011) 123:e368–9. 10.1161/CIRCULATIONAHA.110.004655 21403117

[20] Liao JM Nasseri F Nachiappan AC Kuban J Cheong BY. Left atrial myxoma presenting as a cystic mass. Tex Heart Inst J. (2013) 40:358–9. PMID: 23914040 23914040 PMC3709226

[21] Watanabe H Nara I Yamaura G Iino K Iino T Shimbo M Blood balloon induced by an atrial myxoma in the heart. Circulation. (2014) 130:2351–3. 10.1161/CIRCULATIONAHA.114.010732 25539524

[22] Shabestari MM Fazlinezhad A Moravvej Z Tashnizi MA Azari A Bigdelu L. A case of left atrial myxoma with unusual tumor vascularity. Asian Cardiovasc Thorac Ann. (2015) 23:458–60. 10.1177/0218492313513776 24887916

[23] Suzuki T Hata M Yamaya K Saitou T Haba F Matsuno M Cystic myxoma which obstructed the mitral valve orifice;report of a case. Kyobu Geka. (2020) 73:380–3. PMID: 32398397 32398397

[24] Azad S Dutta N Roy Chowdhuri K Ramman TR Chandra N Radhakrishnan S Atypical left ventricular myxoma: unusual echocardiographic and histopathological features. World J Pediatr Congenit Heart Surg. (2020) 11:NP129–129NP131. 10.1177/2150135117742626 29506452

[25] Halim J van Schaagen FR Riezebos RK Lalezari S. Giant intracardiac blood cyst: assessing the relationship between its formation and previous cardiac surgery. Neth Heart J. (2015) 23:392–4. 10.1007/s12471-015-0707-4 26043925 PMC4497992

